# Survival in a patient with severe paraneoplastic hyponatremia: a case report

**DOI:** 10.1186/1757-1626-1-248

**Published:** 2008-10-17

**Authors:** Gary V Walker, Michael C Peterson

**Affiliations:** 1 BCM-Michael Debakey Center, Room M220, One Baylor Plaza, Houston, TX 77030, USA; 2 Division of General Internal Medicine, University of Utah SOM, Salt Lake City, UT 84648 and Cardiac Hospitalist Service, Central Utah Clinic, Provo, UT 84604, USA

## Abstract

**Background:**

Hyponatremia is a common and potentially life threatening problem in patients with neuroendocrine tumors.

**Case presentation:**

A 54-year-old female with a history of smoking and narcotic dependency presented to her primary care physician with nausea, vomiting, fatigue, malaise, ataxia and a serum sodium of 100 mEq/L. A chest computerized tomography (CT) revealed a 4.1 × 4.9 cm precarinal/pretracheal mass encircling the right brachiocephalic vein. A mediastinal biopsy found a malignant, intermediate-size small cell (oat cell) carcinoma. Saline infusion along with intravenous furosemide successfully corrected her hyponatremia. Unfortunately, the patient later died of complications related to her cancer and cancer therapy.

**Conclusion:**

Paraneoplastic hyponatremia can be severe, but even severe cases may be successfully treated.

## Introduction

Hyponatremia is a common clinical problem that when symptomatic may manifest with nausea, disorientation, seizures, coma, cerebral edema and even death [1]. It is evident in approximately 15% of patients with small-cell lung cancer [2]. The etiology of hyponatremia in small-cell carcinoma has been attributed most often to high levels of vasopressin (AVP); however, atrial natriuetic peptide (ANP) has been implicated as well [3,4]. Correction of hyponatremia is usually successful at moderately low sodium levels, although it must be done slowly to prevent irreversible osmotic demylination. It has been shown that correction is more likely to be successful in persons with higher sodium levels and that death is more likely for persons with more severe hyponatremia [5].

We report a case of very severe hyponatremia related to a neuroendocrine tumor where the patient's sodium was successfully corrected.

## Case presentation

A 54-year-old white female presented to her primary care physician with a four-day history of nausea, vomiting, fatigue, malaise, blurry vision and difficulty walking. She reported a subjective weight loss over the previous month. She had a previous history of major depressive disorder with anxiety, chronic back pain, alcohol dependence in partial remission, 70 pack year smoking history, alcohol withdrawal seizures, hypertension, herniated nucleus pulposus of cervical, thoracic and lumbar spine, cerebrovascular accident at 35 years of age and narcotic dependence. Her past surgical history revealed bilateral mastectomy for fibrocystic disease with reconstructive surgery, total knee replacement, bilateral cataract surgeries, right knee arthroscopy with meniscus repair, appendectomy, tonsillectomy, cervical and lumber spine fusion, as well as vaginal hysterectomy with bilateral salpingo-oopherectomy for non-cancerous reasons at age 36. Her father died of myocardial infarction at age 52, brother died of myocardial infarction and her mother with Paget's disease of the breast. She reported having three sons with two living. Her medications include amlodipine, oxycodone, oxycodone and acetaminophen, diazepam, metoprolol succinate, and aspirin. She reported taking these medicines as prescribed.

One month previously, a blood test showed a serum sodium of 121 mEq/L. The patient left the doctor's office against medical advice, receiving no recommendations for evaluation or therapy. When she returned later with the same symptoms, her serum sodium level was 100 mEq/L. The patient displayed slowed speech and some difficulty with balance and gait. Potassium and creatinine levels were within normal limits. She denied excessive water ingestion or diuretic use. The patient had no evidence of hypothyroidism, heart failure or adrenal insufficiency. Urine osmolality was 413 and serum osmolality was 213. A chest CT showed a 4.1 × 4.9 cm precarinal/pretracheal mass encircling the right brachiocephalic vein.

The patient was treated with gentle saline infusion and low doses of intravenous furosemide with frequent monitoring of electrolytes. Her treatment was later amended to fluid restriction after her sodium rose. On successive days her serum sodium was 113 mEq/L, 125 mEq/L and 132 mEq/L respectively and it was noted that the patient's neurologic symptoms quickly resolved. The patient was discharged on the fifth day with a serum sodium of 133 mEq/L and instructed to restrict free water intake to 900 cc/day. A biopsy at mediastinoscopy revealed a malignant, intermediate-size small cell (oat cell) carcinoma. Radiation and chemotherapy was initiated. Later, the patient unfortunately died from complications related to her cancer and therapy.

The causes of hyponatremia vary widely, from pharmacological to physiological factors. Neuroendocrine tumors may cause hyponatremia. Narcotics have also been implicated as a cause of hyponatremia; one patient recovered from a narcotic-induced hyponatremia of 108 mEq/L [6]. We believe that hyponatremia in this patient was a result of her neuroendocrine tumor perhaps augmented by her use of narcotics.

Serum sodium levels of 100 mEq/L or lower are extremely rare in the literature. One schizophrenic patient with polydipsia died with a serum sodium of 92 mEq/L [7]. Survival has been reported in patients whose serum sodium levels were 86 mEq/L [8], 101 mEq/L [5] and 103 mEq/L [9].

## Conclusion

In general, correction of hyponatremia should be done slowly to avoid osmotic demyelination. However, more rapid correction may be warranted in symptomatic patients, for example those with seizure or coma. For treating severe, symptomatic hyponatremia, infusion of hypertonic saline or saline combined with furosemide, with frequent monitoring of sodium levels, has been recommended. Asymptomatic or mild hyponatremia can be treated with fluid restriction or with demeclocycline (though this is expensive) while a cause and definitive treatment are sought. This paper illustrates two important points: 1) extreme hyponatremia may develop with surprisingly few symptoms if it occurs over a prolonged period of time and 2) even very severe hyponatremia may be successfully corrected – though in this case, the patient had an associated disease process that was later fatal.

## Competing interests

The authors declare that they have no competing interests.

## Authors' contributions

Conception and design (mcp), data abstraction and analysis (both), writing initial draft (gvw), revision and editing (both), approval of final draft (both)

## Consent

Our patient is deceased and thus unable to sign consent for this publication, but did sign consent to be treated during the hospitalization.  All identifying information has been removed.
